# Immunological and molecular insights into acinar-ductal metaplasia and atypical flat lesions as precursor lesions of pancreatic ductal adenocarcinoma

**DOI:** 10.1186/s13046-026-03643-4

**Published:** 2026-01-13

**Authors:** Aslihan Yavas, Leon Boshoven, Kai Horny, Sebastian Haensch, Wolfgang Goering, Martin Schlensog, Lena Haeberle, Irene Esposito

**Affiliations:** 1 Institute of Pathology, University Hospital of Düsseldorf, Heinrich Heine University, Moorenstrasse 5, Düsseldorf, 40225 Germany; 2 Core Unit Bioinformatics, Medical Faculty, Heinrich Heine University, Düsseldorf, Germany; 3 Center for Personalized Medicine Oncology, Medical Faculty, University Hospital of Düsseldorf, Heinrich Heine University, Düsseldorf, Germany; 4 Center for Digital Medicine, Heinrich Heine University, Düsseldorf, Germany; 5 Center for Advanced Imaging, Heinrich Heine University, Düsseldorf, Germany

**Keywords:** Atypical flat lesion, Acinar-ductal metaplasia, Pancreatic cancer precursors, Microenvironment, Genetic alterations

## Abstract

**Background:**

Pancreatic ductal adenocarcinoma (PDAC) is known to develop through a stepwise progression from precursor lesions, such as pancreatic intraepithelial neoplasias (PanIN). An alternative carcinogenic pathway has been proposed via transformation of acinar cells, with development of acinar-ductal metaplasia (ADM) and atypical flat lesions (AFL). Defining the characteristics of PDAC precursors is crucial to better understand PDAC carcinogenesis.

**Methods:**

15 KC (*Ptf1a*^*Cre/+*^, *Kras*^*LSLG12D/+*^) and 15 KPC-like mice (*Ptf1a*^*Cre/+*^, *Kras*^*LSLG12D/+*^, *Trp53*^*LoxP/LoxP*^, referred as fKPC hereafter) were sacrificed at different time points. A meticulous morphological evaluation was performed to define different lesion types. Multiplex immunofluorescence staining was applied to define the characteristics of the immune and stromal microenvironment of the lesions. To investigate the association between the genetic alterations and the components of the microenvironment, all lesion types were subjected to next-generation sequencing (NGS) using a 20 genes-panel.

**Results:**

AFL showed a trend towards a more intense immune cell infiltration compared to PanIN and ADM. AFL had higher number of CD4^+^ helper T cells, FOXP3^+^ regulatory T cells, and CD19^+^ B cells than all other analyzed lesions. They displayed more CD8^+^ cytotoxic T cells and FOXP3^+^ cells than PDAC, while peripheral and central PDAC tissues tended to be infiltrated by macrophages in higher frequency. In addition, αSMA-expressing myofibroblastic cancer-associated fibroblasts were tendentially more frequent in AFL than other lesions. PDAC appeared to have higher CXCL12 expression and more common CD109^+^ cells than other lesions. In NGS analysis, none of the lesions in fKPC mice revealed additional coding mutations, while the preneoplastic lesions in 7 KC mice showed variable coding alterations in 16 different genes. The most frequently affected genes were *Arid1a*,* Rnf43*, and *Pik3ca*. PDAC precursors in KC mice showed more dense infiltration of adaptive immune cells than in fKPC mice, supporting the immunosuppressive role of *Trp53* alterations.

**Conclusions:**

Our study highlights the unique immunological and stromal features of AFL. Moreover, reinforcing their potential as precursor lesions, ADM and AFL exhibit variable alterations in the genes that have a critical role in PDAC carcinogenesis.

**Graphical Abstract:**

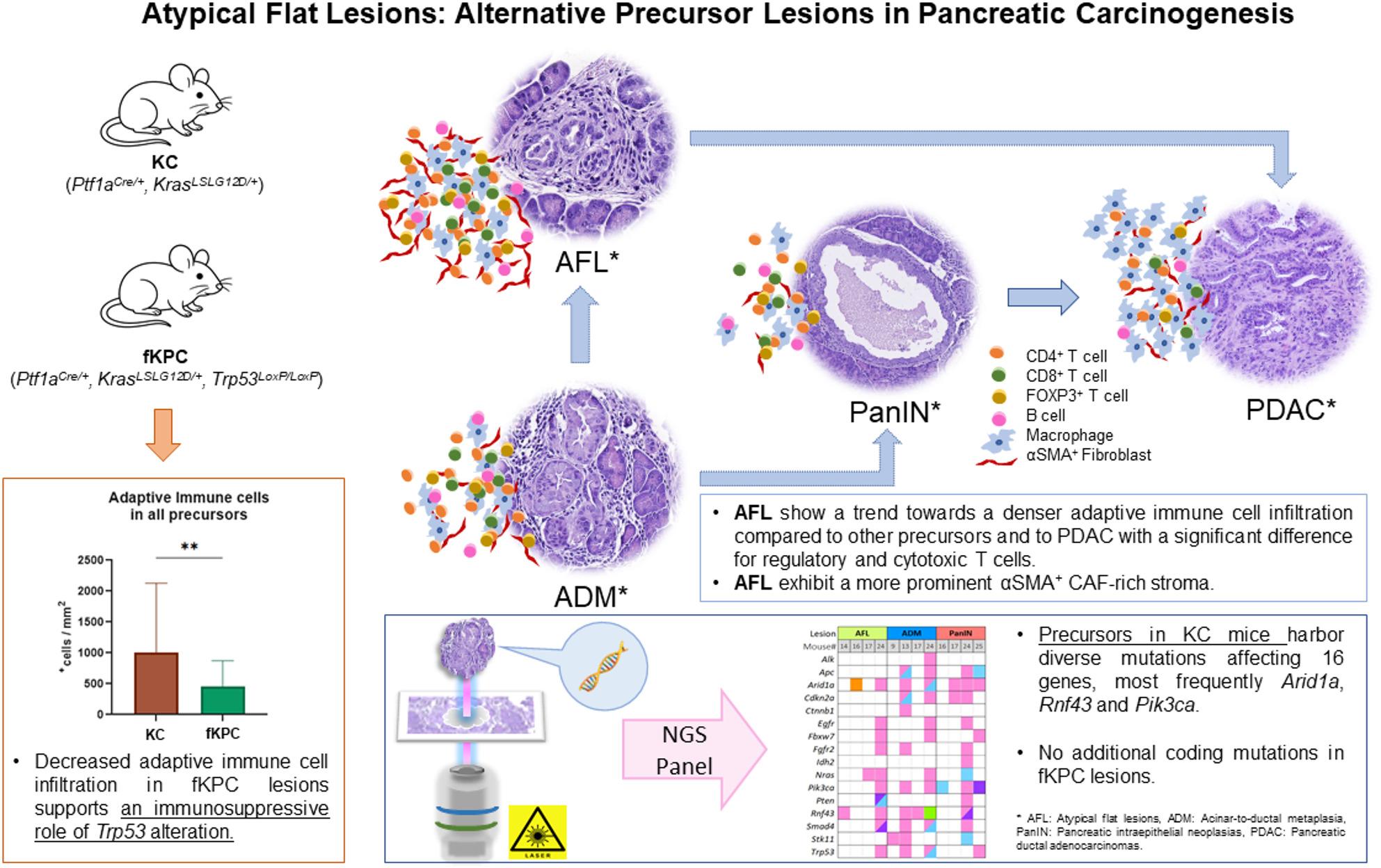

**Supplementary Information:**

The online version contains supplementary material available at 10.1186/s13046-026-03643-4.

## Background

Pancreatic ductal adenocarcinoma (PDAC) is the most common type of pancreatic cancer with an increasing incidence and a 5-year survival rate of 11-13.3% [1, 2]. It is predicted to become the second leading cause of cancer-related deaths by 2030 due to advanced disease at diagnosis and limited treatment options [3, 4].

A stepwise progression model from precursor lesions to carcinoma has been described. The most well-known precursor lesions of PDAC with ductal differentiation are pancreatic intraepithelial neoplasias (PanIN), intraductal papillary mucinous neoplasms (IPMN), and mucinous cystic neoplasms (MCN) [5–7]. Additionally, acinar-ductal metaplasia (ADM) and atypical flat lesions (AFL) arising from acinar cells have been proposed as potential precursor lesions of PDAC in genetically engineered mouse models (GEMMs), and lesions resembling mouse AFL have been identified in patients with familial risk of PDAC [8–11]. PanIN, ADM, and AFL are microscopic lesions in contrast to macroscopically visible IPMN and MCN. PanIN are characterized by flat or papillary architecture with varying degrees of cytological atypia and mucin production [12]. ADM exhibit tubular structures surrounded by fibrosis without significant cytological atypia. Given their origin from acinar cells that undergo metaplasia into ductal cells, ADM can express both acinar and ductal cell markers in early stages. AFL are frequently found in ADM areas and characterized by a flat epithelium with ductal differentiation consisting of atypical cells with surrounding abundant swirl-like stroma [8–10]. Unlike the other PDAC precursors, the biological significance of ADM and AFL in PDAC carcinogenesis is still not well characterized.

PDAC forms an immunosuppressive tumor microenvironment (TME) during carcinogenesis, which is characterized by complex stromal and immunological interactions. Tumor-associated macrophages represent the dominant immune cell population of the TME and play a critical role in the development of PDAC and the progression of its precursor lesions [13–15]. Furthermore, the function of the adaptive immune system cells, including antigen-presenting cells, CD4^+^ helper T cells, and CD8^+^ cytotoxic T cells is often suppressed by the neoplastic cells via various mechanisms, such as the activation of immunosuppressive FOXP3^+^ regulatory T cells and myeloid-derived suppressor cells or the expression of immune checkpoint molecules [16, 17]. Acinar-ductal metaplasia occurs as a reversible event in response to acute pancreatic injury and inflammation, but becomes irreversible in the presence of chronic injury or oncogenic *KRAS* mutation and forms PanIN-like lesions as an initial event of PDAC carcinogenesis [18]. It has been reported that the interactions between acinar cells and infiltrating immune cells, especially macrophages, determines the extent of ADM formation after acute pancreatic injury [13, 19, 20]. Depletion of macrophages in the early regenerative stages resulted in a decrease of ADM development, whereas depletion in the late stages caused a delay of pancreatic repair leading to chronic inflammation [19]. Moreover, it has been shown that innate immune cells such as macrophages and neutrophils promote and maintain acinar trans-differentiation, while adaptive immune cells such as B- and T-cells support normal regeneration after injury by stabilizing the inflammatory environment and limiting the numbers of innate cell populations [21]. There is no detailed data about the immune microenvironment of AFL, except for macrophage accumulation around the lesions [10].

Cancer-associated fibroblasts (CAFs) are an additional significant, heterogeneous component of the TME in PDAC involved in the formation of the desmoplastic stroma and with complex growth- and immune cell-modulating functions [22]. It is known that after pancreatic injury, acinar cells recruit stromal fibroblasts, which in turn play a tumorigenic role with the surrounding macrophages [23, 24]. Moreover, it has been recently reported that CAFs induce acinar to ductal cell trans-differentiation in acinar cells and mouse CAFs co-cultures, suggesting that the CAFs secretome might play a significant role in ADM development and PDAC initiation [25]. Furthermore, AFL, which are frequently found in ADM areas, have been reported to have a special swirl-like stroma composed of αSMA expressing myofibroblastic cancer-associated fibroblasts (myCAFs) [10].

The CXCL12-CXR4 axis has been reported as a key player for the emergence of a tumor-promoting and immunosuppressive TME in PDAC. CXCL12 (C-X-C motif ligand 12) is a chemotactic chemokine, a marker for inflammatory CAFs (iCAFs), has been shown to promote cell survival, proliferation, immune evasion, angiogenesis, and chemoresistance in PDAC via its receptor CXCR4 (C-X-C motif receptor 4). In PDAC, CXCL12 has been reported being expressed primarily by activated fibroblasts in the stroma, while CXCR4 is mainly localized on the tumor cells [26–30]. CXCL12 and its receptors are considered potential therapeutic targets in PDAC; although their expression has been described in PanIN and IPMN [31, 32], no data are available regarding their role in ADM and AFL. In addition, CD109, a glycophosphatidylinositol-binding membrane protein, interacts with TGF-ß1 (transforming growth factor ß1) and EGFR (epidermal growth factor receptor), leading to cell growth, cell differentiation, and epithelial to mesenchymal transition [33, 34]. In PDAC, CD109 can be expressed both in tumor cells and in the surrounding stroma, and it is associated with shorter disease-free and overall survival, tumor progression, and higher rates of distant metastases [35, 36]. CD109 therefore represents a possible prognostic marker for PDAC, but its exact role in PDAC carcinogenesis needs further investigation.

In this study, we aimed at characterizing the role of ADM and AFL as alternative PDAC precursors focusing on the composition of their TME and their genetic landscape.

## Methods

### Collective

The genetically modified mouse collective was maintained at the Central Facility for Animal Research and Scientific Animal Welfare Tasks (ZETT) of the Heinrich Heine University in Düsseldorf. The collective consisting of 15 KC (*Ptf1a*^*Cre/+*^, *Kras*^*LSLG12D/+*^) and 15 KPC-like mice (*Ptf1a*^*Cre/+*^, *Kras*^*LSLG12D/+*^, *Trp53*^*LoxP/LoxP*^, for simplicity referred as fKPC mice) were sacrificed at different time points ranging from 1 to 7 months (Table 1). Harvested pancreas tissues were fixed in formalin and embedded in paraffin after tissue processing. Genotype confirmation was performed by polymerase chain reaction (PCR) analysis [37, 38]. Human formalin-fixed paraffin-emffbedded (FFPE) blocks containing AFL were retrieved from the archive of the Institute of Pathology at the University Hospital Düsseldorf. Tissue sections (1.5 μm) were subjected to hematoxylin-eosin staining and examined meticulously under the light microscope by two experienced pathologists (AY, IE) for lesions’ classification and annotation.

### Laser-captured microdissection and next-generation sequencing

A series of at least 15 FFPE sections with a thickness of 8 μm were generated for laser captured microdissection. Available AFL, ADM, PanIN, and PDAC areas were extracted using the Zeiss Palm Microbeam Laser captured microdissection (LMD) microscope (Carl Zeiss Microscopy GmbH, Jena, Germany). At least 250,000 μm² of total tissue were excised for each lesion type per mouse. Lesions of the same type were pooled across all previously prepared sections. In addition, normal pancreatic tissue was collected from one KC and one fKPC mouse as reference.

DNA isolation from the tissues obtained by LMD was performed using QiAMP^®^ DNA Micro Kit (Qiagen, Hilden, Germany) according to the manufacturer’s instructions. Libraries for the IonTorrent NGS system were created using the Ion AmpliSeq Library Kit 2.0 (ThermoFisher Scientific, Waltham, USA) for a 20 genes panel, which includes 191 hotspots of genes that are relevant for PDAC based on scientific evidence and publicly available mutation databases (Suppl. Table 1). Isolated DNAs were amplified via PCR and stripped to Ion Xpress Barcodes (ThermoFisher Scientific, Waltham, USA). After purification using AMPure XP Beads (Fischer Scientific, Hampton, USA), acquired libraries were quantified using the Ion Library TaqMan Quantitation Kit (ThermoFisher Scientific, Waltham, USA) in StepOnePlus Ultra, Real-Time PCR cycler (ThermoFisher Scientific, Waltham, USA) following the manufacturer’s protocol. DNA-concentration was adjusted to 100 pM to perform massive parallel sequencing using the Ion S5 system with suitable Ion chips and sequencing kits (ThermoFisher Scientific, Waltham, USA). Acquired primary data was checked for quality sufficiency and extracted as FASTQs.

Sequencing data was evaluated using the DNA-Seq-Varlociraptor pipeline (version 3.22.0) [39]. First, raw sequencing data was aligned to the murine reference genome mus musculus GRCm39 using BWA mem software and ensembl (release 108) (version 0.7.17) [40]. Then, candidate variants were called using freebayes (version 1.3.6) [41] and delly (version 1.1) [42] and then processed by Varlociraptor [43]. Varlociraptor is a generic, uncertainty-aware variant caller calculating maximum a posteriori probabilities for complex events. For getting probabilities and evaluating variants, we defined somatic events as following: (1) Non-zero allele frequency (AF) in one preneoplastic lesion, but not the others (2). Non-zero AF in any preneoplastic lesion (3). If tumor sample was available: non-zero AF in tumor but not lesional samples and (4) non-zero AF in tumor and any lesional sample. All events include being absent in the normal pancreas samples, having a resolution of 1% and 5% local false discovery rate (fdr) - control threshold. Variants with an AF below 5% were excluded from the analysis and the remaining variants were annotated using ensembl variant effect predictor (vep) [44]. We investigated the coding variants (defined as those annotated with a HGSVp value) and further filtered to exclude those appearing on only one strand, mutations with low amplicon coverage (< 100x), and variants present in only one of two overlapping amplicons. The variants were manually evaluated and filtered for sequencing artifacts and known sequencing errors typical of semiconductor-based methods, such as frameshifts in base repeats, using Integrative Genomics Viewer (IGV) [45, 46].

### Estimation of DNA copy number variation in human AFL by low-coverage whole-genome sequencing

Seven microdissected human AFL from 3 cases (one with PDAC, two with high-grade precursor lesions without PDAC) were analyzed by low-coverage whole genome sequencing as previously described [47].

Genomic DNA from FFPE samples was amplified using the Ampli1™ WGA kit (Menarini Silicon Biosystems, Bologna, Italy) and purified with SPRIselect beads (Beckman Coulter, Lahntal, Germany). Libraries were prepared using the Ampli1™ low-pass kit (Menarini Silicon Biosystems). The final library concentration was determined on the fragment analyzer (Advanced Analytical technologies, AATI, Ames, Iowa, USA) with the Agilent high sensitivity genomic DNA 50 kb kit (Agilent Technologies, Ratingen, Germany). Sequencing was performed on the Ion S5TM system, and data were mapped to the GRCh37/hg19 reference genome. Ion Reporter software (Version 5.12.0.0) was used to determine copy number variations (CNVs), comparing tumor to normal samples, with regions of log2 ratio > + 0.2 or < -0.2 considered significant. A median of the absolute values of all pairwise differences (MAPD) value < 0.35 was used as a quality threshold.

### Multiplex immunofluorescence staining

After establishment of all antibodies by immunohistochemistry and monoplex immunofluorescence, an Opal-6-plex immunofluorescence multiplex protocol was performed according to manufacturer’s instructions (Suppl. Table 2). The images of the stained multiplex immunofluorescence slides were acquired using a Leica TCS SP8 STED 3X microscope (Leica Microsystems GmbH, Wetzlar, Germany) at the Center for Advanced Imaging (CAI) of the Heinrich Heine University Duesseldorf. Images were captured at 1024 × 1024 pixels and 8-bit depth. Each antibody with its associated fluorophore was recorded in its own sequence, corresponding to the excitation and emission wavelengths of the Opal fluorophores (Suppl. Table 3). Image processing was performed in ImageJ (FIJI, Wayne Rasband, National Institutes of Health and the Laboratory for Optical and Computational Instrumentation, LOCI, University of Wisconsin). For each slide, two pictures per lesion, if possible, were obtained. Afterwards one region of interest (ROI) was defined regarding the tumor microenvironment. Quantification of the positively stained cells was carried out semi-automatically for αSMA, CD4, CD8, CD19, CD20, CD63, CD109, F4/80, and FOXP3 by a “*Fiji*” macro. In brief, the macro applies a 20-pixel radius “*rolling ball background subtraction*” to the channel of choice, slightly adjusts the dynamic range of the image, thresholds it using an autothreshold algorithm as “*Mean*”, removes single pixel events by the “*despecle*”-function, and separates fused regions by a simple “*watershed*” processing. Areas were then counted by the “*analyze particle function*” and detailed readout results (count and area) were used for downstream analysis. The Fiji-macro, exemplary input and result data are provided at: https://github.com/SHaensch/2025_F4I80Quant. To account for both tissue area and total cell density, immune cell counts were normalized by dividing the number of positive immune cells both by the ROI size (in mm²) and the total number of DAPI^+^ cells within that ROI [48, 49]. This double normalization controls for variations in both sample size and cellularity, providing a standardized measure of immune cell presence per unit area and per cell. CXCL12 and CXCR4 were evaluated separately for epithelium and stroma using the immunoreactivity score (IRS), which measures the amount of positively stained cells (graded 0–4) and the staining intensity (graded 0–3). The IRS is calculated by multiplying these two values (IRS = Intensity score × Percentage score); resulting in a total score between 0 and 12. The intensity score was validated by comparing it with the mean gray value derived from selected regions of interest using the Analyze function with suitable Macro in ImageJ.

### Statistical evaluation

Statistical analyses were carried out using GraphPad Prism 8 (GradPad Software Inc., San Diego, USA) independently for each single marker by comparing across multiple lesion groups. Data sets were tested for normality. For comparisons of 2 groups, Student t test or Mann-Whitney-U test was used. More than 2 groups were compared with each other using one-way ANOVA or Kruskal-Wallis test. The results were presented as median values and interquartile ranges (IQR: Q1-Q3). P values ​​less than 0.05 were considered statistically significant (* *p* < 0.05, ** *p* < 0.01, *** *p* < 0.001).

## Results

**Fig. 1:**
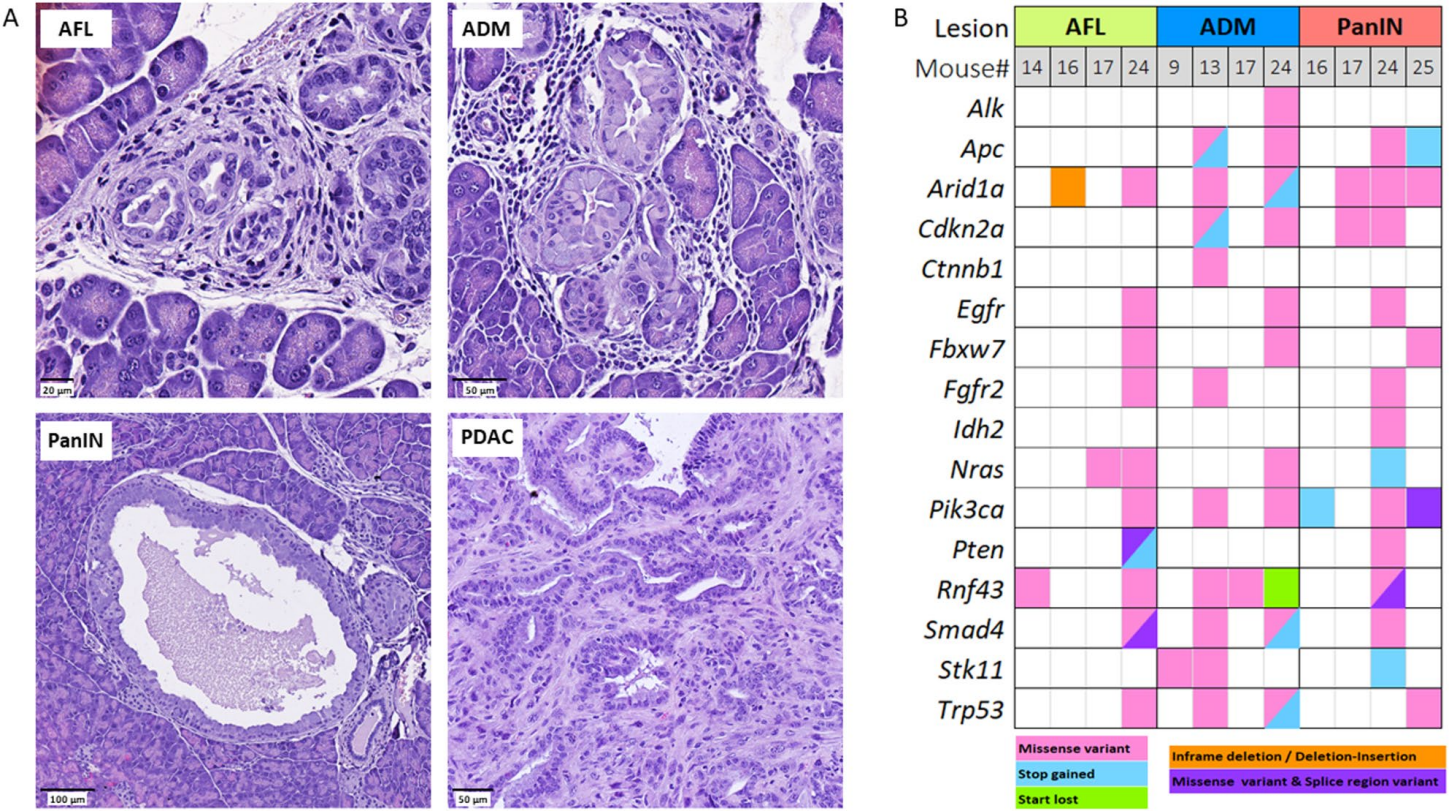
**A** Representative Hematoxylin-Eosin images for AFL, ADM, PanIN, and PDAC. Scale bars represent 20 μm, 50 μm, 100 μm and 50 μm, respectively. **B** Overview of coding variant consequences summarized for 16/20 examined genes by next generation sequencing for each KC mouse and lesion type

A careful morphological evaluation was performed on hematoxylin-eosin-stained slides that were generated from 15 KC and 15 fKPC mice and all identified lesion types were recorded for each mouse. AFL, ADM, and PanIN were observed in all KC mice, however none of them had progressed to PDAC. In contrast, all but one of the fKPC mice had already developed PDAC within 2 months. In 10 fKPC mice, the full spectrum of the investigated lesions was detected, whereas in 4 only ADM and PDAC were identified, and one mouse developed AFL, ADM, and PanIN (Table 1; Fig. 1A).

**Table 1:** Detailed information about the mouse cohort

Mouse number	Mouse genotype	Sex	Age (months)	AFL	ADM	PanIN	PDAC
9	*Ptf1a*^*Cre/+*^, *Kras*^*LSLG12D/+*^	F	2	+	+	+	-
19	*Ptf1a*^*Cre/+*^, *Kras*^*LSLG12D/+*^	M	2	+	+	+	-
21	*Ptf1a*^*Cre/+*^, *Kras*^*LSLG12D/+*^	M	3	+	+	+	-
24	*Ptf1a*^*Cre/+*^, *Kras*^*LSLG12D/+*^	F	3	+	+	+	-
25	*Ptf1a*^*Cre/+*^, *Kras*^*LSLG12D/+*^	M	3	+	+	+	-
4	*Ptf1a*^*Cre/+*^, *Kras*^*LSLG12D/+*^	M	4	+	+	+	-
6	*Ptf1a*^*Cre/+*^, *Kras*^*LSLG12D/+*^	F	4	+	+	+	-
7	*Ptf1a*^*Cre/+*^, *Kras*^*LSLG12D/+*^	F	4	+	+	+	-
17	*Ptf1a*^*Cre/+*^, *Kras*^*LSLG12D/+*^	F	4	+	+	+	-
1	*Ptf1a*^*Cre/+*^, *Kras*^*LSLG12D/+*^	F	5	+	+	+	-
3	*Ptf1a*^*Cre/+*^, *Kras*^*LSLG12D/+*^	F	5	+	+	+	-
11	*Ptf1a*^*Cre/+*^, *Kras*^*LSLG12D/+*^	F	7	+	+	+	-
13	*Ptf1a*^*Cre/+*^, *Kras*^*LSLG12D/+*^	M	7	+	+	+	-
14	*Ptf1a*^*Cre/+*^, *Kras*^*LSLG12D/+*^	M	7	+	+	+	-
16	*Ptf1a*^*Cre/+*^, *Kras*^*LSLG12D/+*^	M	7	+	+	+	-
8	*Ptf1a*^*Cre/+*^, *Kras*^*LSLG12D/+*^, *Trp53*^*LoxP/LoxP*^	M	1	+	+	+	-
20	*Ptf1a*^*Cre/+*^, *Kras*^*LSLG12D/+*^, *Trp53*^*LoxP/LoxP*^	F	1	+	+	+	+
22	*Ptf1a*^*Cre/+*^, *Kras*^*LSLG12D/+*^, *Trp53*^*LoxP/LoxP*^	F	1	+	+	+	+
23	*Ptf1a*^*Cre/+*^, *Kras*^*LSLG12D/+*^, *Trp53*^*LoxP/LoxP*^	F	1	+	+	+	+
29	*Ptf1a*^*Cre/+*^, *Kras*^*LSLG12D/+*^, *Trp53*^*LoxP/LoxP*^	M	1	+	+	+	+
2	*Ptf1a*^*Cre/+*^, *Kras*^*LSLG12D/+*^, *Trp53*^*LoxP/LoxP*^	M	2	+	+	+	+
5	*Ptf1a*^*Cre/+*^, *Kras*^*LSLG12D/+*^, *Trp53*^*LoxP/LoxP*^	F	2	+	+	+	+
10	*Ptf1a*^*Cre/+*^, *Kras*^*LSLG12D/+*^, *Trp53*^*LoxP/LoxP*^	F	2	-	+	-	+
12	*Ptf1a*^*Cre/+*^, *Kras*^*LSLG12D/+*^, *Trp53*^*LoxP/LoxP*^	F	2	+	+	+	+
15	*Ptf1a*^*Cre/+*^, *Kras*^*LSLG12D/+*^, *Trp53*^*LoxP/LoxP*^	F	2	+	+	+	+
18	*Ptf1a*^*Cre/+*^, *Kras*^*LSLG12D/+*^, *Trp53*^*LoxP/LoxP*^	F	2	+	+	+	+
26	*Ptf1a*^*Cre/+*^, *Kras*^*LSLG12D/+*^, *Trp53*^*LoxP/LoxP*^	M	2	-	+	-	+
27	*Ptf1a*^*Cre/+*^, *Kras*^*LSLG12D/+*^, *Trp53*^*LoxP/LoxP*^	M	2	-	+	-	+
28	*Ptf1a*^*Cre/+*^, *Kras*^*LSLG12D/+*^, *Trp53*^*LoxP/LoxP*^	F	2	-	+	-	+
30	*Ptf1a*^*Cre/+*^, *Kras*^*LSLG12D/+*^, *Trp53*^*LoxP/LoxP*^	F	2	+	+	+	+

### Molecular landscape of AFL and ADM

Pooled DNA extracted from 96 microdissected lesions, including 26 AFL, 30 ADM, 26 PanIN, and 14 PDAC from 15 KC and 15 fKPC mice together with two normal pancreas samples were subjected to targeted next-generation sequencing. We assessed somatic variants in either a specific or any preneoplastic lesion and in 7/15 KC mice (47%) we found 106 different coding variants in 16/20 sequenced gene hotspots (Suppl. Table 4). In contrast, we found no additional coding alterations in fKPC mice that were somatic to the PDAC or any preneoplastic lesion.

4/15 (26.6%) AFL, 4/15 (26.6%) ADM, and 4/15 (26.6%) PanIN were found to have coding variants in 16/20 genes (Fig. 1B). Excluding *Kras*, the most frequently affected genes were *Arid1a*,* Rnf43*, and *Pik3ca*, followed by *Trp53*,* Smad4*,* Nras*,* Apc*,* Cdkn2a*, *Stk11*, while *Alk*,* Ctnnb1*,* Egfr*,* Fbxw7*,* Fgfr2*,* Idh2*, and *Pten* gene alterations were rare. Coding *Braf*,* Gnas*, and *Idh1* gene alterations were not found (Table 2; Fig. 1B).

**Table 2:** Number and frequency of affected mice with an altered gene, respectively, shown for each lesion type and each gene investigated with next generation sequencing

KC mice (*n* = 15)	Number / frequency (%) of mice with altered genes per lesion type		
Gene	AFL	ADM	PanIN
*Arid1A*	2 (13.3%)	2 (13.3%)	3 (20%)
*Alk*	0 (0%)	1 (6.6%)	0 (0%)
*Apc*	0 (0%)	2 (13.3%)	2 (13.3%)
*Braf*	0 (0%)	0 (0%)	0 (0%)
*Cdkn2a*	0 (0%)	2 (13.3%)	2 (13.3%)
*Ctnnb1*	0 (0%)	1 (6.6%)	0 (0%)
*Egfr*	1 (6.6%)	1 (6.6%)	1 (6.6%)
*Fbxw7*	1 (6.6%)	1 (6.6%)	1 (6.6%)
*Fgfr2*	1 (6.6%)	1 (6.6%)	1 (6.6%)
*Gnas*	0 (0%)	0 (0%)	0 (0%)
*Idh1*	0 (0%)	0 (0%)	0 (0%)
*Idh2*	0 (0%)	0 (0%)	1 (6.6%)
*Kras*	15 (100%)	15 (100%)	15 (100%)
*Nras*	2 (13.3%)	1 (6.6%)	1 (6.6%)
*Pik3ca*	1 (6.6%)	2 (13.3%)	3 (20%)
*Pten*	1 (6.6%)	0 (0%)	1 (6.6%)
*Rnf43*	2 (13.3%)	3 (20%)	1 (6.6%)
*Smad4*	1 (6.6%)	2 (13.3%)	1 (6.6%)
*Stk11*	0 (0%)	2 (13.3%)	1 (6.6%)
*Trp53*	1 (6.6%)	2 (13.3%)	1 (6.6%)

AFL in mouse 14, 16, and 17 revealed alterations in only one gene; i.e., *Rnf43*,* Arid1a* or *Nras*, respectively. In contrast, AFL in mouse 24 showed alterations in multiple genes, also found in ADM and PanIN of the same mouse. Similar to mouse 24, ADM in mouse 13 was characterized by alterations in several genes, in contrast to ADM of mouse 9 with only *Stk11* and ADM of mouse 17 with only *Rnf43* alterations (Fig. 1B). Variants of a given gene were mostly missense mutations and unique for the single lesion type.

### Analysis of immune and stromal microenvironment of AFL and ADM

In this study, a total of 198 regions of interest (ranging from 7861 to 338512 µm^2^ in size) including normal pancreas, AFL, ADM, PanIN, peripheral (pPDAC), and central PDAC (cPDAC) were meticulously examined by the multiplex immunofluorescence method; in detail, 101 ROIs were analyzed for the expression of CD4, CD8, FOXP3, CD19, F4/80 markers, and 97 ROIs for the expression of αSMA, CK19, CD109, CXCR4, and CXCL12.

**Fig. 2:**
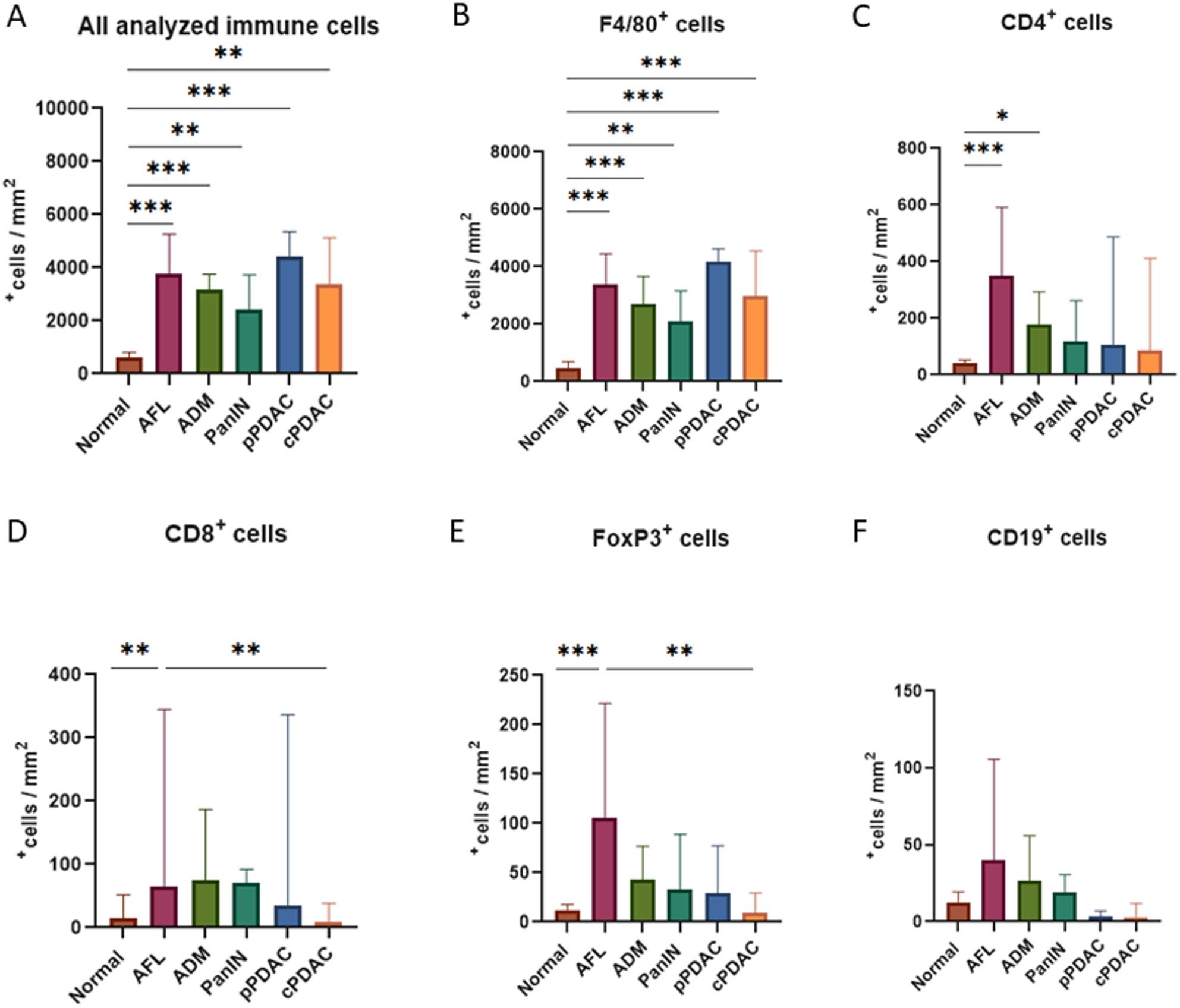
**A** Normalized immune cell counts to the size of ROIs (mm^2^) by multiplex immunofluorescence. AFL show a tendency towards a denser immune cell infiltration among precursor lesions. **B**-**F** Detailed analysis of the immune cells with F4/80, CD4, CD8, FOXP3, and CD19 markers in normal tissue, PDAC, and its precursors. Kruskal-Wallis test, *p < 0.05, **p < 0.01, ***p < 0.001

Compared to the normal pancreas, all the analyzed lesion types revealed higher number of immune cell infiltration (*p* < 0.0001, Fig. 2A). More importantly, the microenvironment of AFL tended to be infiltrated more densely by immune cells (median 3785^+^cells/mm^2^, IQR: 2579–5283) than that of the other precursor lesions. While pPDAC appeared to be associated with the highest level of immune cell infiltration in the surrounding tissue (median 4437^+^cells/mm^2^, IQR: 2656–5171), cPDAC showed a tendency for lower immune cell infiltration than AFL (median 3389^+^cells/mm^2^, IQR: 2184–4659) (Fig. 2A).

The detailed phenotypical analysis revealed that precursor lesions had higher accumulation of adaptive immune cells in their TME than PDAC (precursors: median 60^+^cells/mm^2^, IQR: 21–190 vs. PDAC: median 17^+^cells/mm^2^, IQR: 4–61, *p* < 0.0001). Macrophages were the dominant population of the investigated immune cell types in all lesions and in the normal tissue and represented the largest proportion of all analyzed immune cells and all DAPI^+^ cells in PDAC compared to precursor lesions (*p* = 0.007) (Table 3).

**Table 3:** Distribution of the analyzed immune cells in precursor lesions and PDAC as median^+^cells/mm^2^ and IQR: Q1-Q3. IQR: interquartile range. *p* < 0.05 is significant

Marker	Precursors	PDAC	*p*-values
F4/80	2832, IQR: 1326–3805	3753, IQR: 2492–4517	*p* = 0.007
CD4	197, IQR: 68–468	92, IQR: 27–397	*p* = 0.15
CD8	74, IQR: 34–189	19, IQR: 9–39	*p* = 0.0005
FOXP3	44, IQR: 22–106	19, IQR: 4–47	*p* = 0.003
CD19	21, IQR: 0–63	3, IQR: 0–6	*p* = 0.002

^+^cells/mm^2^ and IQR: Q1-Q3

**Fig. 3:**
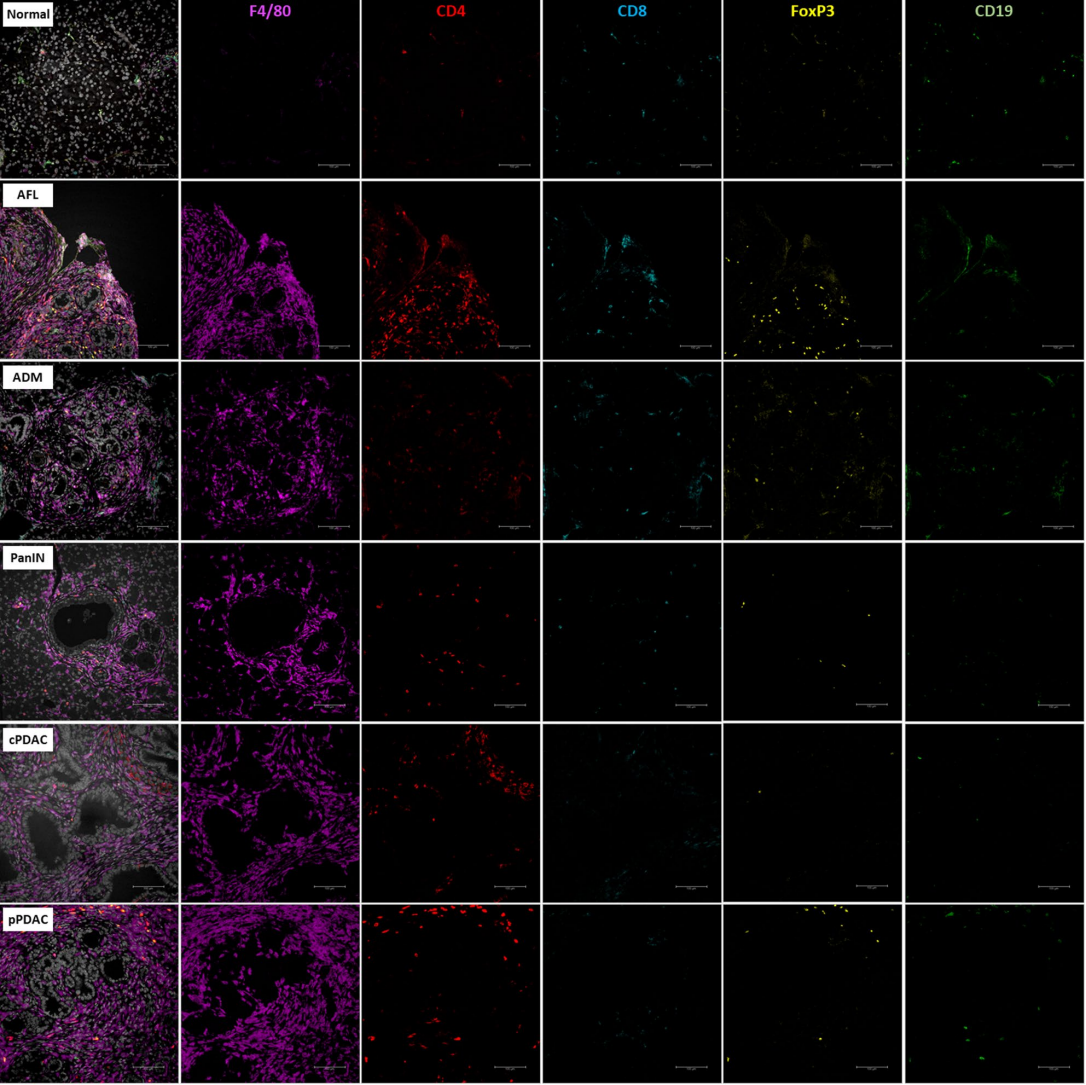
Representative images of multiplex immunofluorescence staining for F4/80, CD4, CD8, FOXP3, and CD19 markers. Scale bars represent 100 μm

Compared to other lesions, AFL showed a trend towards a higher number of macrophages in the surrounding area than ADM and PanIN (median 3373, 2690, 2105^+^cells/mm^2^, IQR: 1474–4582, 2134–3775, 707–3396, respectively), but slightly less than PDAC (median 3753^+^cells/mm^2^, IQR: 2492–4517) (Figs. 2B and 3). Moreover, AFL appeared to harbor higher number of CD4^+^ helper T cells (median 349^+^cells/mm^2^, IQR: 81–614), FOXP3^+^ regulatory T cells (AFL median 106^+^cells/mm^2^, IQR: 39–228 vs. cPDAC median 9^+^cells/mm^2^, IQR: 3–23, *p* < 0.01), and CD19^+^ B cells (median 40^+^cells/mm^2^, IQR: 0-107) than all analyzed lesion types, and also displayed higher number of CD8^+^ cytotoxic T cells (median 14^+^cells/mm^2^, IQR: 40–107) compared to PDAC with a significant difference in comparison with cPDAC (median 9^+^cells/mm^2^, IQR: 8–34, *p* = 0.005) (Figs. 2C-F and 3). In addition, ADM and PanIN revealed higher number of CD4^+^ helper T cells, CD8^+^ cytotoxic T cells, FOXP3^+^ regulatory T cells, and CD19^+^ B cells than PDAC with no statistical significance (Figs. 2C-F and 3). Similar trends were observed when normalizing by dividing the number of positive immune cells by the total number of DAPI^+^ cells (Suppl. Figure 1). These results highlight the existence of an immune active microenvironment in precursor lesions, particularly in AFL, which is different from the immunosuppressive microenvironment in PDAC.

**Fig. 4:**
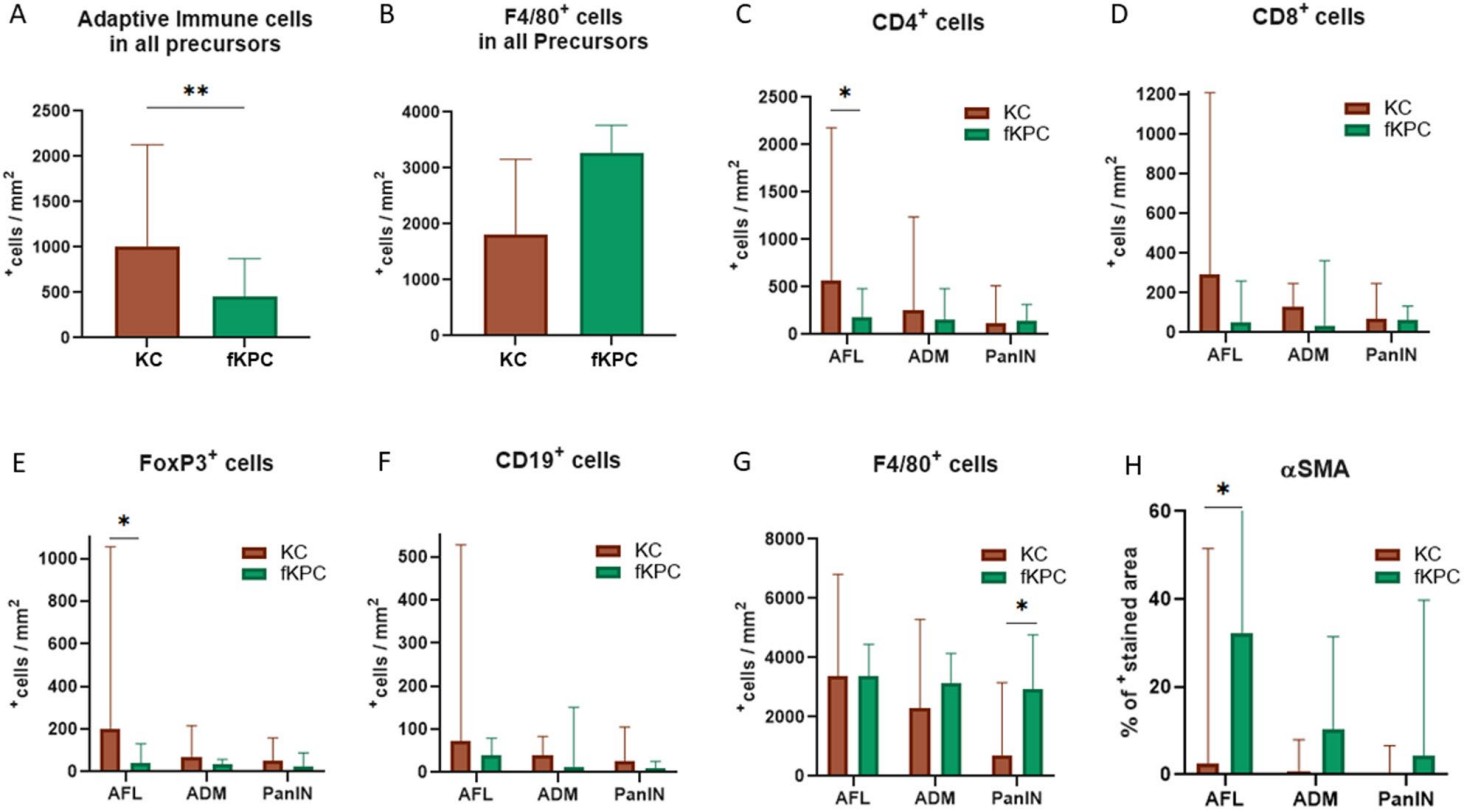
Comparison of the immune cell distribution based on normalized immune cell counts to the size of ROIs (mm^2^) and αSMA stained areas in AFL, ADM, and PanIN of the KC and fKPC mice by multiplex immunofluorescence. Mann-Whitney U test, two-way ANNOVA, *p < 0.05, **p < 0.01

Interestingly, the precursor lesions in KC mice revealed higher number of adaptive immune cells compared to those in fKPC mice (KC: median 707^+^cells/mm^2^, IQR: 296–963 vs. fKPC: 339^+^cells/mm^2^, IQR: 123–595, *p* = 0.008). This trend was consistent across all precursor subtypes and individual adaptive immune cell types, with significant differences observed in AFL for CD4^+^ helper T cells (*p* = 0.02) and FOXP3^+^ regulatory T cells (*p* = 0.03) (Fig. 4A and C-F). In contrast, the precursor lesions in fKPC mice showed a tendency for denser macrophage accumulation compared to KC mice (KC: median 1810^+^cells/mm^2^, IQR: 741–3642 vs. fKPC: median 3276^+^cells/mm^2^, IQR: 2524–3827, *p* = 0.06) with a significant increase in PanIN (*p* = 0.01) (Fig. 4B and G).

**Fig. 5:**
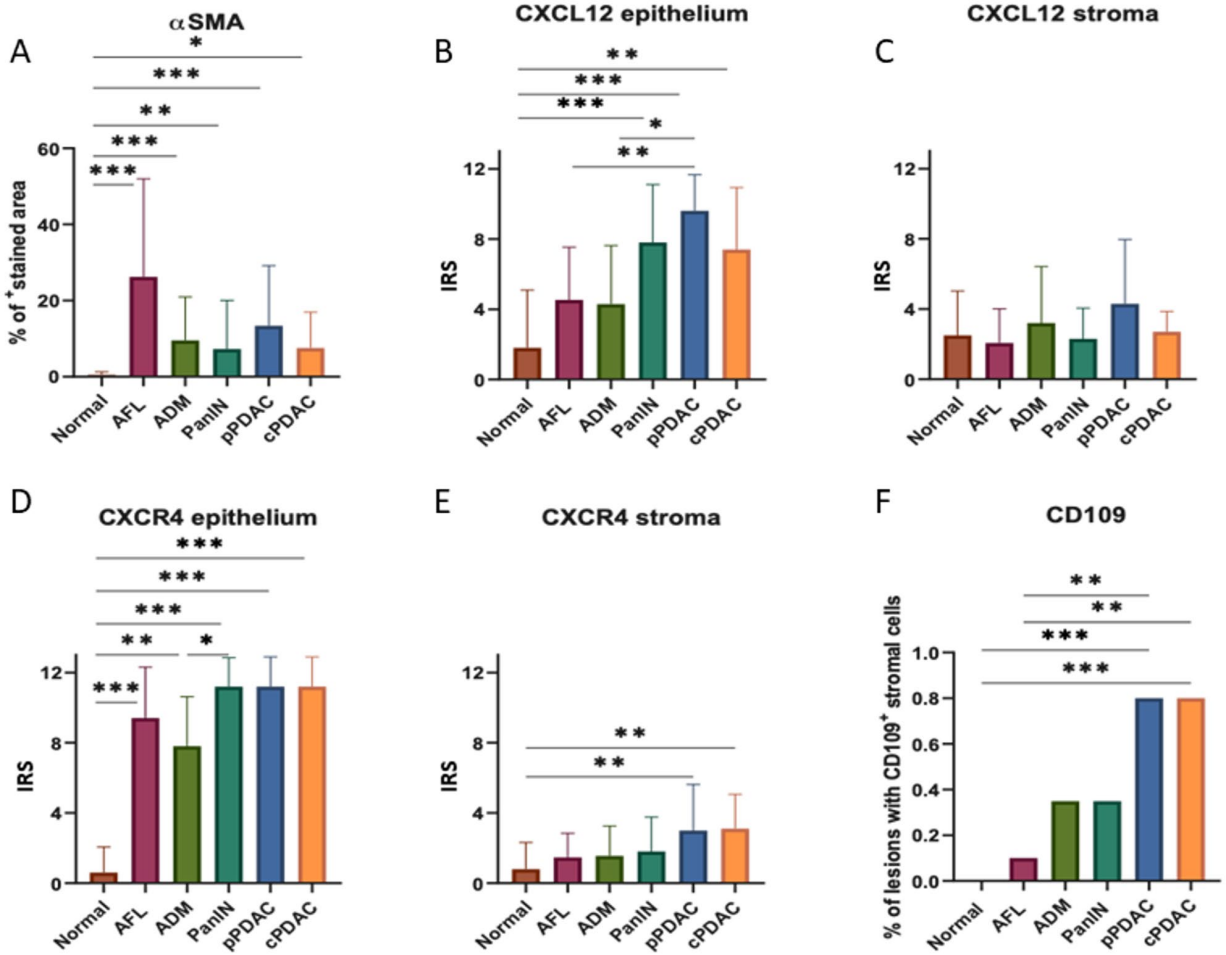
**A** Comparison of αSMA stained areas in normal tissue, PDAC, and its precursors by multiplex immunofluorescence. **B**-**E** CXCL12 and CXCR4 expression analysis in epithelium and stromal tissue. **F** Frequency of the lesions with CD109^+^ cells in surrounding tissue. Kruskal-Wallis test, *p < 0.05, **p < 0.01, ***p < 0.001. IRS: Immune reactivity score

**Fig. 6:**
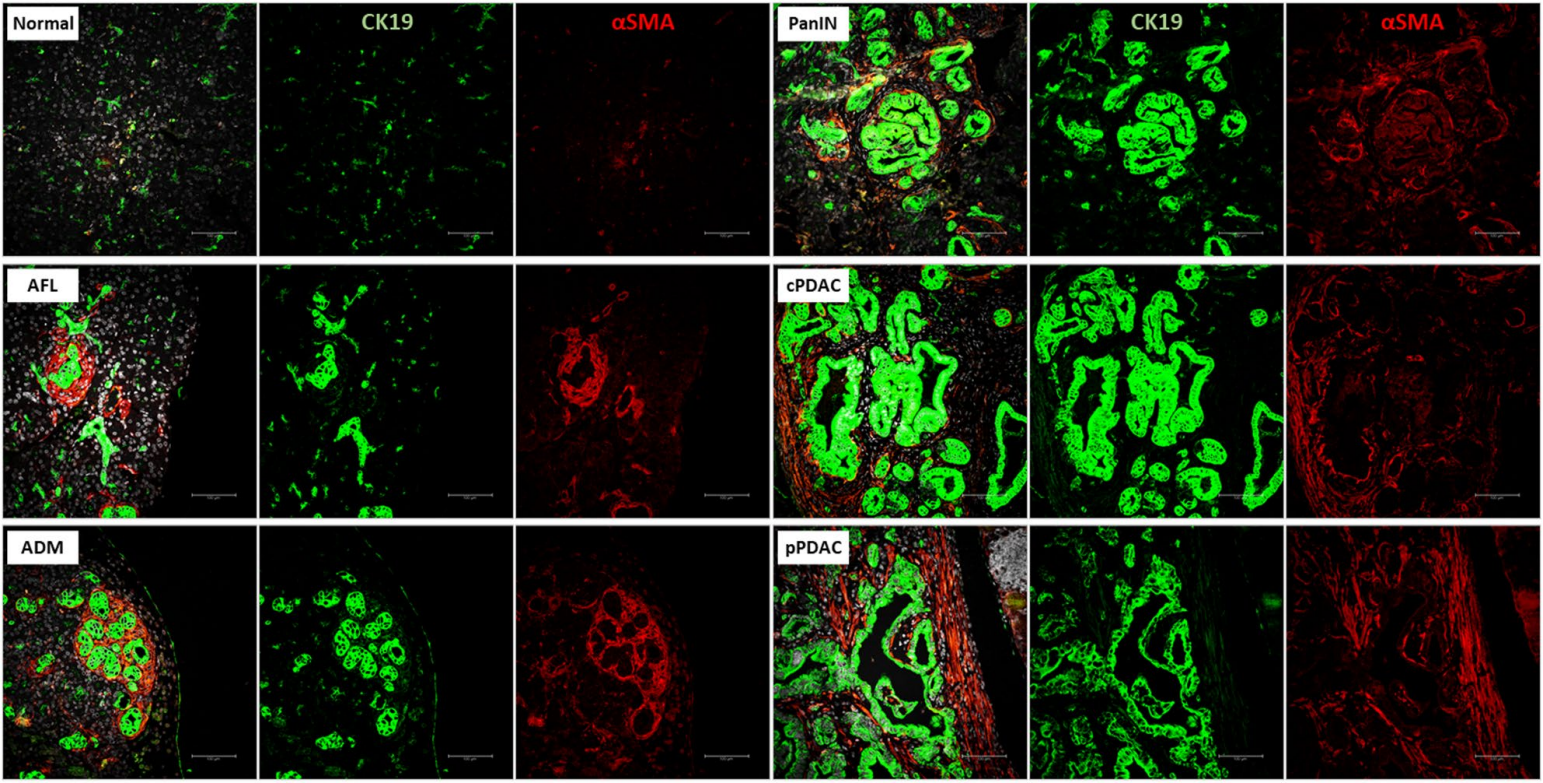
Representative images of multiplex immunofluorescence staining for αSMA and CK19. Scale bar represents 100 μm

**Fig. 7:**
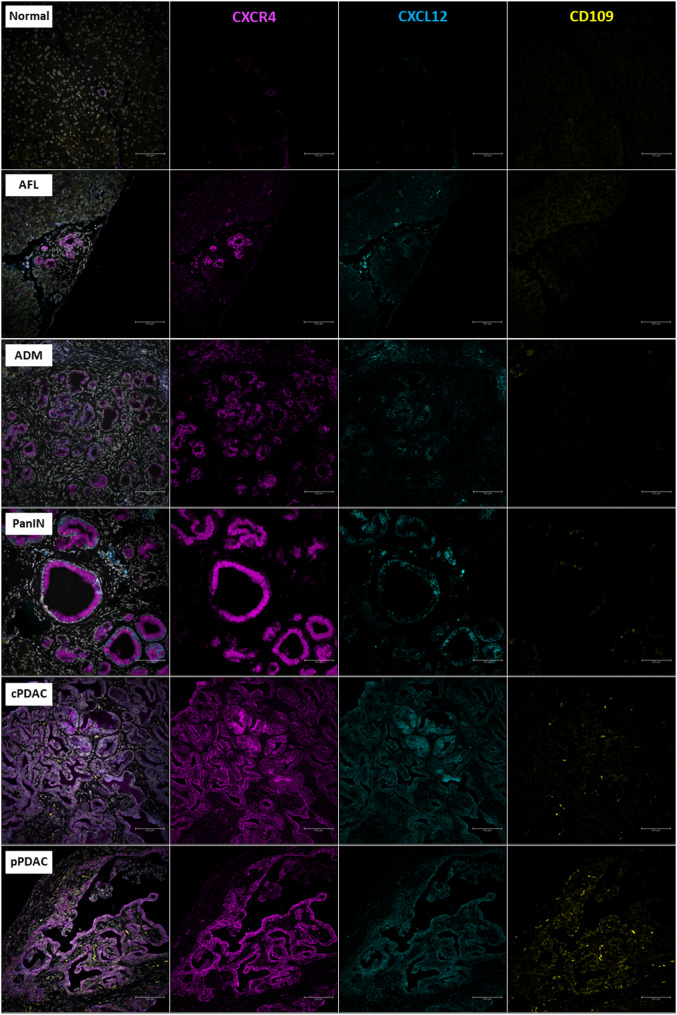
Representative images of multiplex immunofluorescence staining for CXCL12, CXCR4, and CD109 markers. Scale bars represent 100 μm

All precursor lesions and PDAC demonstrated significantly higher amount of αSMA^+^ stromal cells than the normal pancreas (median 0.1% of ROI, IQR: 0.04–0.6, *p* < 0.0001, Fig. 5A). More importantly, the peculiar “swirling” stroma of AFL tended to show the highest amount of αSMA expression level (median 22% of ROI, IQR: 2.4–50.3) followed by ADM (median 7.3% of ROI, IQR: 0.7–11.2) (Figs. 5A and 6). Precursor lesions in fKPC mice showed significantly higher expression of αSMA in their TME than in KC mice (median 5.9% vs. 0.8% of ROI, IQR: 0.02–24.4 vs. 0.1–3.6, respectively, *p* < 0.001, Fig. 4H). Consistently, high expression levels of CXCL12 were not detected in AFL stroma, indicating a predominance of αSMA^+^ myCAFs over iCAFs. Both CXCL12 and CXCR4 displayed distinct expression profiles across pancreatic lesions supporting their immunosuppressive and tumor promoting role in PDAC progression. The CXCR4 and CXCL12 expression in epithelial cells tended to be lower in AFL and ADM (CXCR4: both median IRS = 8 and CXCL12: both median IRS = 4) than PanIN, pPDAC, and cPDAC (CXCR4: all median IRS = 12 and CXCL12: all median IRS ≥ 7), with a significant difference compared to pPDAC for CXCL12 expression (pPDAC median IRS = 8 vs. AFL and ADM, *p* = 0.02 and *p* = 0.008, respectively, Fig. 5B and D). In the stromal compartment, both CXCR4 and CXCL12 levels showed tendency for increase in PDAC (median IRS ≥ 1.5 and median IRS = 3, respectively) compared to precursor lesions (median IRS = 1 and median IRS ≤ 2, respectively) with no statistical significance (Figs. 5C and E and 7). In addition, stromal cells of the precursor lesions showed less frequent CD109 expression than PDAC, supporting its tumorigenic contribution (80% of PDAC with CD109^+^ cells vs. 27% of precursors with CD109^+^ cells, *p* < 0.0001) (Figs. 5F and 7).

#### AFL in human samples

**Fig. 8:**
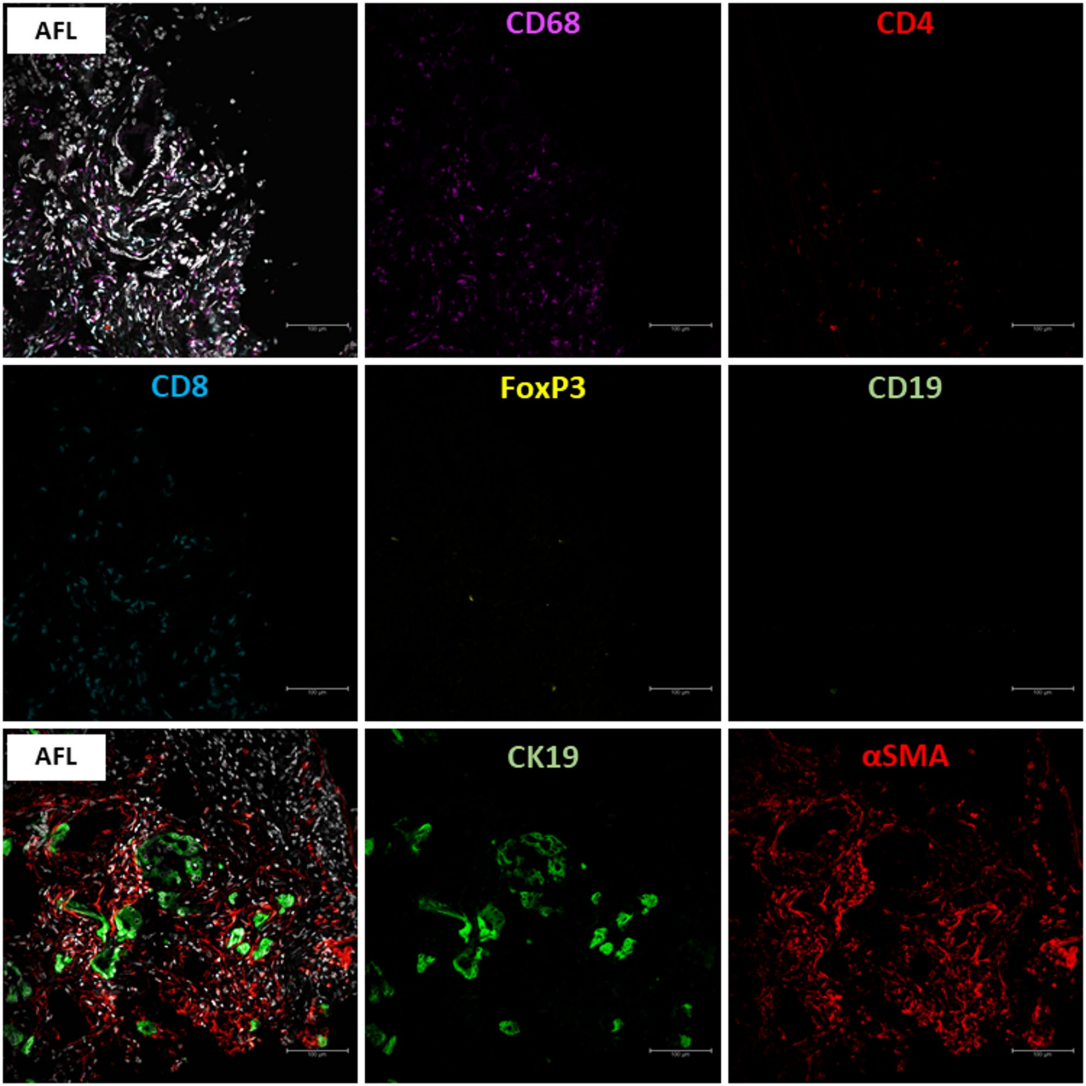
Immune cell infiltration, CK19 and αSMA expression in human AFL by multiplex immunofluorescence. Scale bars represent 100 μm

Three available human AFL samples were also subjected to multiplex immunofluorescence staining. Similar to mice, AFL in human pancreas tissue was characterized by an increased immune cell infiltration (AFL mean 2263 cells/mm^2^ vs. normal mean 254 cells/mm^2^) with macrophage predominance (44% of all immune cells) and increased αSMA expression compared to normal tissue (AFL mean 10.6% of the ROI vs. normal mean 0.1% of the ROI) (Fig. 8). Due to the small number of the cases, statistical analysis could not be performed.

We therefore formulated the hypothesis that AFL may represent a site of genetic instability, leading to expression of neoantigens that induce a very early immune response. To test this hypothesis, we performed CNV analysis in 7 AFL from 3 cases, which revealed recurrent alterations in 4 lesions obtained from one case with PDAC. Other 3 AFL from 2 different cases without PDAC (one with multiple high grade PanIN and the other with high grade IPMN) demonstrated no alterations. We then compared these results with those we previously generated in PanIN lesions [47] and found that recurrent CNV were more frequent in human AFL than PanIN lesions (Suppl. Figure 2, Suppl. Table 5).

## Discussion

PanIN, IPMN, and MCN are phenotypically ductal lesions and represent PDAC precursors [5–7]. However, increasing evidence in recent studies, particularly using genetically engineered mouse models, suggests the possibility of an alternative carcinogenic pathway, with ADM and AFL as putative PDAC precursors [8–10, 20, 50–53].

A progression model originating in the centroacinar-acinar compartment and resulting in the development of PanIN-like lesions was proposed by our group after a meticulous analysis of 92 pancreatic resection specimens from individuals affected by PDAC and benign conditions, such as chronic pancreatitis and serous cystic neoplasms [9]. In following studies, a direct progression model, which originates from ADM and leads to the development of AFL and PDAC, thus by-passing the “mucinous” PanIN pathway, was suggested [8, 10]. AFL are atypical tubular structures with enlarged, hyperchromatic nuclei, surrounded by a fibrous and cellular stroma in KC/KPC mice and patients with a familial predisposition for PDAC [10].

Our comprehensive analysis underscores differences in immune infiltration, stromal composition, and genetic alterations across pancreatic precursor lesions, with a particular emphasis on AFL. These differences offer valuable insights into the early pancreatic tumorigenesis process.

Consistent findings in both mouse models and human tissue samples reveal that AFL are characterized by a highly active immune microenvironment. In AFL, we showed a trend towards a higher level of infiltration by macrophages and adaptive immune cells, including CD4^+^ helper T cells, CD8^+^ cytotoxic T cells, and FOXP3^+^ regulatory T cells, than that observed in the other precursors. In a recent study, innate immune cells, including macrophages and neutrophils, have been identified as playing a pivotal role in the process of acinar trans-differentiation. Conversely, adaptive immune cells, including B- and T-cells, have been shown to facilitate regeneration following injury by stabilizing the inflammatory environment and constraining the proliferation of innate cell populations [21]. The observed trend towards a decrease in the proportion of adaptive immune cells and an increase in the proportion of macrophages from AFL-ADM-PanIN to PDAC suggests that AFL and ADM may be still under the influence of the regulatory mechanisms of the adaptive immune system against acinar dedifferentiation stimuli of innate immune cells. The relatively elevated immune presence in AFL and other precursor lesions, suggests that these early lesions may exist in a dynamic immune-active state that may represent an opportunity for immune-mediated tumor suppression or clearance.

The stromal compartment of AFL also displayed remarkable features, characterized by abundant αSMA^+^ myCAFs forming a “swirling” stromal pattern unique to these lesions. This pattern is associated with a low expression of CXCL12 (and its receptor CXCR4), a chemokine known to be expressed by iCAFs that facilitates immunosuppression and tumor progression in PDAC [26–31]. In line with the recent studies, our findings suggest that the emergence of CXCL12 expressing immunosuppressive iCAFs may occur in the advanced stages of the PDAC carcinogenesis, while the αSMA^+^ myCAFs activation could be an early event already occurring in the AFL as an initial precursor lesion [22, 54]. Despite CXCL12 is classically described as a stroma-derived chemokine, consistent with our findings, epithelial CXCL12 expression has also been documented in mouse PanIN and PDAC lesions [31, 55]. In addition, human studies show variable epithelial CXCL12 levels in PDAC cell lines, tumor cells in PDAC tissue and PanIN [29, 31, 56–58]. Together, these data suggest that both stromal and epithelial CXCL12 signaling play a role in PDAC carcinogenesis, with relative expression depending on tumor stage and microenvironmental context. Additionally, the lower stromal expression of CD109 in precursor lesions compared to PDAC suggests a gradual stromal activation correlating with tumorigenic progression [35, 59]. Collectively, these stromal characteristics point toward a microenvironment in AFL and other early lesions that may support immune engagement rather than suppression.

Importantly, this immunological profile was mirrored in the limited number of human AFL samples examined by multiplex immunofluorescence. Although statistical analyses were constrained by the small number of cases, human AFL appeared to have an increased immune cell infiltration with a predominance of macrophages and a marked increase in αSMA^+^ stromal cells. These findings support the possible translational relevance of the murine data and suggest that the immune activation observed in AFL may be conserved across species.

The TME of PDAC and its precursor lesions intricately gets shaped by specific genetic alterations that drive carcinogenesis and modulate stromal and immune interactions. Among the most frequently altered genes in PDAC are *KRAS*,* TP53*,* CDKN2A*, and *SMAD4*, which not only initiate tumorigenesis but also exert profound influence on the cellular composition and functional state of the TME. It has been shown that oncogenic *KRAS* signaling leads to tumor cell proliferation, fibroblast activation, macrophage infiltration, and subsequently acinar cell dedifferentiation [60, 61]. Additionally, inhibition of active *KRAS* in PDAC leads to more immunoreactive macrophages and a decrease of myeloid derived suppressor cells in the TME [62]. On the other hand, alterations of *TP53*, particularly missense mutations, have been linked to reduced infiltration of cytotoxic CD8⁺ T cells and the upregulation of fibrosis-associated gene programs, contributing to an immunosuppressive microenvironment [63]. In accordance with these findings, we observed that in precursor lesions such as ADM and AFL, the presence of *Trp53* mutation is associated with reduced infiltration of adaptive immune cells, accompanied by increased expression of αSMA^+^ in the surrounding stroma. These findings suggest that *TP53* mutations may not only drive tumorigenesis but also facilitate stromal remodeling and immune evasion at early stages.

Beyond the four canonical drivers, PDAC harbors less common but recurrent mutations in genes such as *RNF43*, *ARID1A*, *TGFβR2*, *GNAS*, and *PIK3CA* [64]. Based on the degree of dysplasia, similar genetic alterations have been detected also in PanIN [65, 66]. While previous studies have reported alterations in *KRAS*,* CDKN2A*, and overexpression of p53 in ADM and AFL [10], our study is the first to systematically investigate the most common PDAC-associated genes of these alternative precursor lesions using an expanded targeted sequencing panel on meticulously microdissected samples.

In the KC mouse model, we identified coding somatic variants in 16 of 20 commonly altered PDAC-associated genes across AFL, ADM, and PanIN lesions. The most frequently mutated genes were *Arid1a*,* Rnf43*, and *Pik3ca*, followed by *Trp53*,* Smad4*,* Nras*,* Apc*,* Cdkn2a*, and *Stk11*. The detected genetic alterations in AFL and ADM support the hypothesis that these lesions, like PanIN, may be legitimate alternative precursors to PDAC. In contrast, lesions from fKPC mice showed no additional coding somatic mutations, suggesting that early, concurrent activation of *KRAS* and *TP53* may be sufficient to drive carcinogenesis in this model. These findings emphasize the importance of temporal and contextual factors in shaping both the genetic evolution and the tumor microenvironment.

Building on these findings, we hypothesized that AFL may represent sites of genetic instability that generate neoantigens, thereby triggering an early immune response. Supporting this, CNV analysis in human AFL revealed recurrent alterations in more samples compared to PanIN lesions, suggesting that AFL may be genetically more unstable. As precursor lesions progress to highly genomic instable PDAC, the TME undergoes alterations that facilitate immune evasion [54, 67–69]. Therefore, AFL may represent a transitional stage where genomic instability may be sufficient to elicit immune responses, whereas advanced PDAC develops mechanisms to suppress these responses despite ongoing genomic alterations.

This study has limitations related to the use of a small gene panel for sequencing, thus not enabling a comprehensive analysis of the genome of ADM and AFL. NGS of laser-captured microdissected tissue allows genetic analysis of lesions that are too small for manual dissection and reduces contamination with non-lesional tissue. However, due to the very low amount of dissected tissue obtained from each single lesion, it was required to pool the same type of lesions, limiting the assessment of genetic heterogeneity. In the TME characterization, we focused on the most relevant players of TME biology; nevertheless, a further characterization of the immune cell subtypes with additional biomarkers is necessary to better understand their exact role in the progression model. Furthermore, the number of human AFL included in the study is too low to draw any final conclusion about the role in pancreatic carcinogenesis and larger collectives are necessary to confirm our observations.

## Conclusions

In conclusion, while PanIN, IPMN, and MCN are established precursor lesions of PDAC, emerging evidence indicates an alternative pathway originating from the centroacinar-acinar compartment that may also progress to PDAC. Our study highlights the distinct immunological and stromal characteristics of AFL with significant immune cell infiltration and “swirling” stroma. Additionally, both ADM and AFL share key mutations with PanIN and PDAC, supporting their potential roles as precursor lesions. Taken together, these findings highlight the contributions of ADM and AFL to PDAC carcinogenesis, underscored by their unique microenvironments, expression profiles, and early genetic alterations.

## Supplementary Information

Supplementary Material 1: Supplementary Table 1: Next generation sequencing panel, which includes 20 genes and 191 hotspots of the genes that are relevant for PDAC based on bioinformatics databases

Supplementary Material 2: Supplementary Table 2: Information about the antibodies used for multiplex immunofluorescence staining. Supplementary Table 3: Antibody panels and Opal fluorophores with excitation and emission wavelengths.

Supplementary Material 3: Supplementary Table 4: Detailed NGS analysis results.

Supplementary Material 4: Supplementary Table 5: Details of the copy number variation analysis in AFL and PanIN (47). AFL: Atypical flat lesion, PanIN: Pancreatic intraepithelial neoplasia, CNA: Copy number alteration. The PanIN section was reproduced based on the original publication data under the Creative Commons Attribution 4.0 (CC BY 4.0) license. The original data was used for table creation. © Author(s) (or their employer(s)) 2023. Re-use permitted under CC BY 4.0. License link: https://creativecommons.org/licenses/by/4.0/

Supplementary Material 5: Supplementary Figure 1: A. Normalized immune cell counts to the total DAPI+ cell number by multiplex immunofluorescence. AFL tended to show the densest immune cell infiltration in precursor lesions. B-F. Detailed analysis of the immune cells with F4/80, CD4, CD8, FOXP3, and CD19 markers in normal tissue, PDAC, and its precursors. Kruskal-Wallis test, *p<0.05, **p<0.01, ***p<0.001.

Supplementary Material 6: Supplementary Figure 2: Copy number variation analysis in AFL and PanIN lesions by low-coverage whole genome sequencing. A. CNVs were detected in 4 out of 7 AFL samples (57%). B. CNVs were present in 2 out of 11 PanIN samples (18%). Red color indicates copy number gains and blue represents copy number losses (47). Panel B was reproduced from the original publication under the Creative Commons Attribution 4.0 (CC BY 4.0) license. No changes were made. © Author(s) (or their employer(s)) 2023. Re-use permitted under CC BY 4.0. License link: https://creativecommons.org/licenses/by/4.0/ .

## Data Availability

All data generated or analyzed during this study are included in this published article and its supplementary information files.

## Abbreviations

PDAC: Pancreatic ductal adenocarcinoma
PanIN: Pancreatic intraepithelial neoplasia
IPMN: Intraductal papillary mucinous neoplasm
MCN: Mucinous cystic neoplasm
ADM: Acinar-ductal metaplasia
AFL: Atypical flat lesion
TME: Tumor microenvironment, immune and stromal microenvironment of the lesions
GEMM: Genetically engineered mouse models
KC: *Ptf1a*^Cre/+^, Kras^LSLG12D/+^ mice
fKPC: *Ptf1a*^Cre/+^, Kras^LSLG12D/+^, Trp53^LoxP/LoxP^mice
NGS: Next-generation sequencing
CAFs: Cancer-associated fibroblasts
myCAFs: Myofibroblastic cancer-associated fibroblasts
iCAFs: Inflammatory cancer-associated fibroblasts
CXCR4: C-X-C motif receptor 4
CXCL12: C-X-C motif ligand 12
TGF-ß1: Transforming growth factor ß1
EGFR: Epidermal growth factor receptor
PCR: Polymerase chain reaction
FFPE: Formalin-fixed paraffin-embedded
LMD: Laser captured microdissection
ROI: Region of interest
IRS: Immunoreactivity score
