# Supplementary material for: Immunological and molecular insights into acinar-ductal metaplasia and atypical flat lesions as precursor lesions of pancreatic ductal adenocarcinoma
Source: J Exp Clin Cancer Res. 2026 Jan 13;45:24. doi: 10.1186/s13046-026-03643-4 (PMC12849142; doi:10.1186/s13046-026-03643-4)
Supplement: Supplementary file 2 — Supplementary Material 2: Supplementary Table 2: Information about the antibodies used for multiplex immunofluorescence staining. Supplementary Table 3: Antibody panels and Opal fluorophores with excitation and emission wavelengths. [file 13046_2026_3643_MOESM2_ESM.docx]

**Supplementary Table 2.** Information about the antibodies used for multiplex immunofluorescence staining.

| **Antibody (Clone)** | **Target** | **IgG** | **Dilution** | **Buffer** | **Target cells** | **Manifacturer** |
| --- | --- | --- | --- | --- | --- | --- |
| **αSMA (1A4)** | Mouse / Human | Mouse | 1:200 | TE / pH9 | Activated stromal cells | Abcam, Cambridge (UK) |
| **CD4 (EPR19514)** | Mouse | Rabbit | 1:75 | TE / pH9 | Helper T cells | Abcam, Cambridge (UK) |
| **CD4 (EP204)** | Human | Mouse | 1:75 | TE / pH9 | Helper T cells | DCS, Hamburg (DE) |
| **CD8α (D4W2Z)** | Mouse | Rabbit | 1:200 | TE / pH9 | Cytotoxic T cells | Cell Signaling Technology, Danvers (USA) |
| **CD8 (C8/144B)** | Human | Mouse | 1:200 | TE / pH9 | Cytotoxic T cells | DAKO Agilent Technologies, Santa Clara (USA) |
| **CD19 (D4V4B)** | Mouse | Rabbit | 1:400 | TE / pH9 | B cells | Cell Signaling Technology, Danvers (USA) |
| **CD20cy (L26)** | Human | Mouse | 1:40 | TE / pH9 | B cells | DAKO Agilent Technologies, Santa Clara (USA) |
| **CD68 (KP1)** | Human | Mouse | 1:100 | TE / pH9 | Macrophages | DAKO Agilent Technologies, Santa Clara (USA) |
| **CD109** **(C9)** | Mouse / Human | Mouse | 1:50 | TE / pH9 | Membrane-binding Glycoprotein | Santa Cruz Biotechnology, Dallas (USA) |
| **CK19 (EP1580Y)** | Mouse / Human | Rabbit | 1:200 | TE / pH9 | Pancreatic duct cells | Abcam, Cambridge (UK) |
| **CXCL12 (4G10)** | Mouse / Human | Mouse | 1:250 | TE / pH9 | Chemokine | Santa Cruz Biotechnology, Dallas (USA) |
| **CXCR4** **(P-159X)** | Mouse / Human | Mouse | 1:100 | TE / pH9 | Chemokine-Receptor | Santa Cruz Biotechnology, Dallas (USA) |
| **F4/80 (D2S9R)** | Mouse | Rabbit | 1:1000 | TE / pH9 | Macrophages | Cell Signaling Technology, Danvers (USA) |
| **FoxP3 (D6O8R)** | Mouse | Rabbit | 1:600 | TE / pH9 | Regulatory T cells | Cell Signaling Technology, Danvers (USA) |
| **FoxP3 (235A/E7)** | Human | Mouse | 1:600 | TE / pH9 | Regulatory T cells | Abcam, Cambridge (UK) |

**Supplementary Table 3.** Antibody panels and Opal fluorophores with excitation and emission wavelengths.

| **Immune Panel Mouse** | **Immune Panel Human** | **Epithelial-Stromal Panel Mouse & Human** | **Opal^TM^-Fluorophor** | **Excitation (nm)** | **Detection ranges (nm)** |
| --- | --- | --- | --- | --- | --- |
| CD19 | CD20 | CK19 | Opal^TM^ 540 | 523 | 534-542 |
| CD4 | CD4 | αSMA | Opal^TM^ 520 | 494 | 511-529 |
| CD8 | CD8 | CXCL12 | Opal^TM^ 690 | 676 | 721-735 |
| F4/80 | CD68 | CXCR4 | Opal^TM^ 570 | 550 | 568-573 |
| FoxP3 | FoxP3 | CD109 | Opal^TM^ 620 | 588 | 607-612 |
| DAPI | DAPI | DAPI | DAPI | 358 | 420-467 |
