# Supplementary figures and images for: Immunological and molecular insights into acinar-ductal metaplasia and atypical flat lesions as precursor lesions of pancreatic ductal adenocarcinoma
Source: J Exp Clin Cancer Res. 2026 Jan 13;45:24. doi: 10.1186/s13046-026-03643-4 (PMC12849142; doi:10.1186/s13046-026-03643-4)

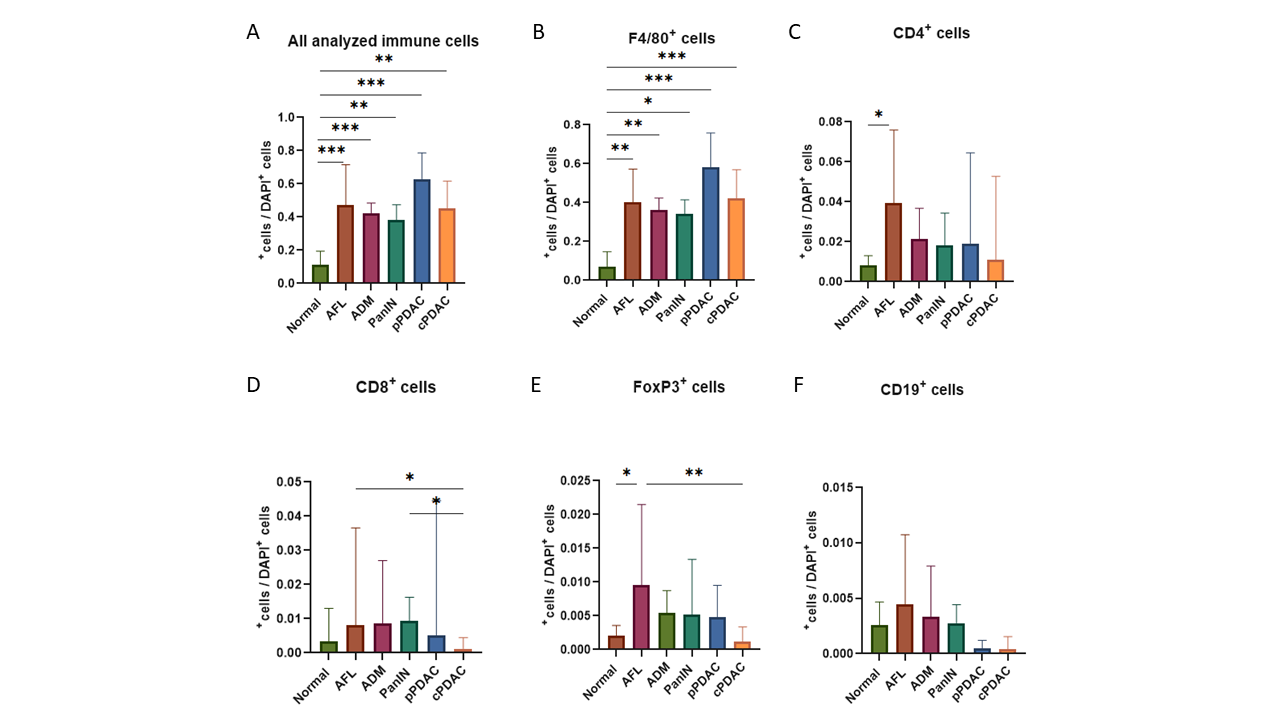

Supplement: Supplementary file 5 — Supplementary Material 5: Supplementary Figure 1: A. Normalized immune cell counts to the total DAPI+ cell number by multiplex immunofluorescence. AFL tended to show the densest immune cell infiltration in precursor lesions. B-F. Detailed analysis of the immune cells with F4/80, CD4, CD8, FOXP3, and CD19 markers in normal tissue, PDAC, and its precursors. Kruskal-Wallis test, *p<0.05, **p<0.01, ***p<0.001. [file 13046_2026_3643_MOESM5_ESM.tif]

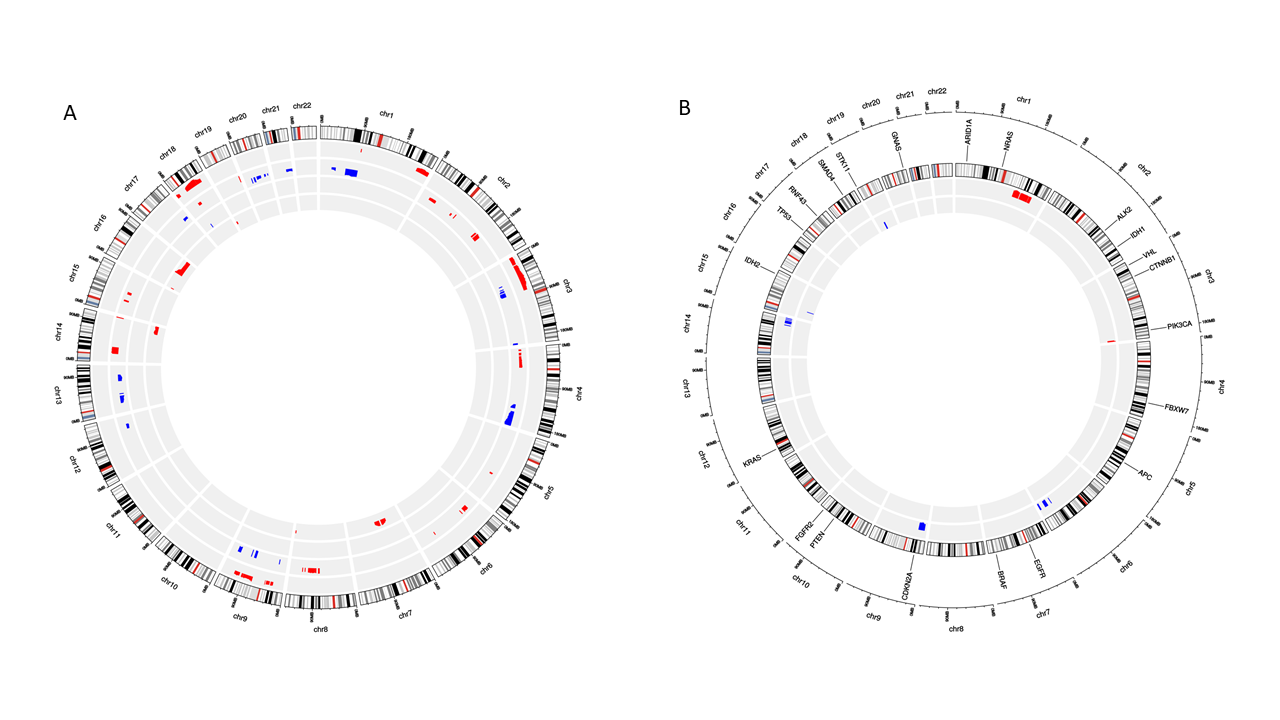

Supplement: Supplementary file 6 — Supplementary Material 6: Supplementary Figure 2: Copy number variation analysis in AFL and PanIN lesions by low-coverage whole genome sequencing. A. CNVs were detected in 4 out of 7 AFL samples (57%). B. CNVs were present in 2 out of 11 PanIN samples (18%). Red color indicates copy number gains and blue represents copy number losses (47). Panel B was reproduced from the original publication under the Creative Commons Attribution 4.0 (CC BY 4.0) license. No changes were made. © Author(s) (or their employer(s)) 2023. Re-use permitted under CC BY 4.0. License link: https://creativecommons.org/licenses/by/4.0/ . [file 13046_2026_3643_MOESM6_ESM.tif]
